# Tafazzin deficiency causes substantial remodeling in the lipidome of a mouse model of Barth Syndrome cardiomyopathy

**DOI:** 10.3389/fmmed.2024.1389456

**Published:** 2024-04-29

**Authors:** Malte Hachmann, Güntas Gülcan, Ranjithkumar Rajendran, Marcus Höring, Gerhard Liebisch, Akash Bachhuka, Michael Kohlhaas, Christoph Maack, Süleyman Ergün, Jan Dudek, Srikanth Karnati

**Affiliations:** ^1^ Institute of Anatomy and Cell Biology, University of Würzburg, Würzburg, Germany; ^2^ Department of Medical Biochemistry, Faculty of Medicine, Atlas University, Istanbul, Turkey; ^3^ Experimental Neurology, Department of Neurology, Justus Liebig University, Giessen, Germany; ^4^ Institute of Clinical Chemistry and Laboratory Medicine, University Hospital of Regensburg, Regensburg, Germany; ^5^ Department of Electronics, Electric, and Automatic Engineering, Rovira I Virgili University, Tarragona, Spain; ^6^ Department of Translational Research, Comprehensive Heart Failure Center, University Hospital Würzburg, Würzburg, Germany; ^7^ Medical Clinic 1, University Hospital Würzburg, Würzburg, Germany

**Keywords:** Barth Syndrome, heart failure, tafazzin, lipids, lipidome, electrospray ionization-tandem mass spectrometry (ESI-MS/MS)

## Abstract

Barth Syndrome (BTHS) is a rare X-linked disease, characterized clinically by cardiomyopathy, skeletal myopathy, neutropenia, and growth retardation. BTHS is caused by mutations in the phospholipid acyltransferase tafazzin (Gene: TAFAZZIN, TAZ). Tafazzin catalyzes the final step in the remodeling of cardiolipin (CL), a glycerophospholipid located in the inner mitochondrial membrane. As the phospholipid composition strongly determines membrane properties, correct biosynthesis of CL and other membrane lipids is essential for mitochondrial function. Mitochondria provide 95% of the energy demand in the heart, particularly due to their role in fatty acid oxidation. Alterations in lipid homeostasis in BTHS have an impact on mitochondrial membrane proteins and thereby contribute to cardiomyopathy. We analyzed a transgenic TAFAZZIN-knockdown (TAZ-KD) BTHS mouse model and determined the distribution of 193 individual lipid species in TAZ-KD and WT hearts at 10 and 50 weeks of age, using electrospray ionization tandem mass spectrometry (ESI-MS/MS). Our results revealed significant lipid composition differences between the TAZ-KD and WT groups, indicating genotype-dependent alterations in most analyzed lipid species. Significant changes in the myocardial lipidome were identified in both young animals without cardiomyopathy and older animals with heart failure. Notable alterations were found in phosphatidylcholine (PC), phosphatidylethanolamine (PE), lysophosphatidylethanolamine (LPE), lysophosphatidylcholine (LPC) and plasmalogen species. PC species with 2–4 double bonds were significantly increased, while polyunsaturated PC species showed a significant decrease in TAZ-KD mice. Furthermore, Linoleic acid (LA, 18:2) containing PC and PE species, as well as arachidonic acid (AA, 20:4) containing PE 38:4 species are increased in TAZ-KD. We found higher levels of AA containing LPE and PE-based plasmalogens (PE P-). Furthermore, we are the first to show significant changes in sphingomyelin (SM) and ceramide (Cer) lipid species Very long-chained SM species are accumulating in TAZ-KD hearts, whereas long-chained Cer and several hexosyl ceramides (HexCer) species accumulate only in 50-week-old TAZ-KD hearts These findings offer potential avenues for the diagnosis and treatment of BTHS, presenting new possibilities for therapeutic approaches.

## 1 Introduction

BTHS is a rare, X-linked, recessive, life-threatening disorder (Barth et al., 1983; Spencer et al., 2006; Dudek and Maack, 2017). It primarily affects males, and is clinically characterized by cardiomyopathy, skeletal myopathy, neutropenia, growth delay, and 3-methylglutaconic aciduria (Clarke et al., 2013; Dudek and Maack, 2017). A recent review has estimated the prevalence of BTHS as 1 case per million males (Taylor et al., 2022). BTHS patients experience a high infant mortality rate due to progressive cardiomyopathy (CM) and a compromised immune system (Dudek and Maack, 2017).

BTHS is caused by loss-of-function mutations in the TAFAZZIN gene (TAZ), located on the distal portion of chromosome Xq28, which encodes tafazzin (Jefferies, 2013; Dudek and Maack, 2017). TAFAZZIN encodes a phospholipid acyltransferase that is involved in the remodeling of CL. CL is located predominantly in the inner mitochondrial membrane and is comprised of two phosphatidylglyceride backbone molecules connected by glycerol (Neuwald, 1997; Bowron et al., 2013). Under physiological conditions, premature CL is de-acylated to monolysocardiolipin (MLCL) and then converted to mature CL by tafazzin. TAFAZZIN mutations in BTHS and consequent inactivation of mitochondrial tafazzin cause a deficiency in the formation of mature CL forms and an increase in intermediate CL types such as MLCL (Vreken et al., 2000; Schlame et al., 2002). The increased MLCL/CL ratio is often used as a diagnostic marker for BTHS (Houtkooper et al., 2009). CL acquires its biochemical properties by the specific composition of its fatty acid sidechains and this determines CL-protein interaction (Lewis and McElhaney, 2009; Planas-Iglesias et al., 2015). With four esterified fatty acids, a highly diversified CL pool is present in most mammalian tissues. Interestingly, in the heart the predominant pattern of CL is tetralinoleyl CL, CL (18:2)_4_, while other tissues exhibit a broader acylation pattern (Schlame et al., 2002; Oemer et al., 2020). The crowded protein environment due to the large amounts of respiratory chain complexes in cardiac mitochondria was found to be responsible for this specified pool of CL species (Schlame et al., 2012; Xu et al., 2019). Tafazzin deficiency may not only impair the homeostasis of the appropriate tissue specific CL pool. Different lipid species are interconnected by a complex network of interdependencies. Notably, recent findings indicate that Tafazzin deficiency may also affect other lipid species, but the extent of shifts in other lipid pools remains unresolved (Kimura et al., 2018; Bozelli and Epand, 2022).

The heart, being one of the most energy-demanding organs in the human body, relies on mitochondria as its primary source of energy in cardiac tissue. The heart muscle has a high mitochondrial density, occupying about 35% of the cardiomyocyte volume, to meet its energy demand (Dudek and Maack, 2017). CL plays a role in various aspects of mitochondrial biology. CL is a wedge-shaped molecule and therefore plays an essential role in shaping mitochondrial morphology by introducing membrane bends. Thereby, it is involved in the formation of the lamellar crista architecture and affects fission and fusion (Bustillo-Zabalbeitia et al., 2014; Stepanyants et al., 2015). CL externalization during mitochondrial stress forms a signaling platform, recruiting the autophagic machinery to damaged mitochondria. The mitophagy adaptor microtubule-associated protein 1A/1B-light chain (LC3) is recruited to mitochondria via its interaction with CL and thereby controls the elongation of the phagophore membrane (Chu et al., 2013). Furthermore, CL is involved in the structure and function of mitochondrial membrane proteins such as the electron transport chain complexes, metabolite carrier proteins and the mitochondrial calcium uniporter (Pfeiffer et al., 2003; Duncan et al., 2018; Bertero et al., 2021). Alterations in the respiratory chain and defects in mitochondrial Ca^2+^ handling synergize to an energetic deficit and oxidative stress in BTHS (Bertero et al., 2021).

A mouse model, in which TAFAZZIN gene expression is systemically reduced, due to the expression of an interfering shRNA (TAZ-KD), is currently the most used animal model for BTHS in the field. The TAZ-KD mice closely resemble the phenotype of BTHS patients. Left ventricular ejection fraction (LVEF) moderately deteriorated between 10 and 15 weeks of age and then remained stable over the time of 50 weeks (Rigaud et al., 2013; Kang et al., 2016; Thompson et al., 2016; Bertero et al., 2021). Hemodynamic measurements and force measurements using isolated cardiomyocytes revealed diastolic dysfunction due to elevated myofilament Ca^2+^ affinity and slowed crossbridge cycling (Watkins et al., 2011). This phenotype resembles BTHS patients, who initially present with dilated cardiomyopathy (DCM), and/or left ventricular non-compaction (Spencer et al., 2006; Roberts et al., 2012; Rigaud et al., 2013; Kang et al., 2016; Thompson et al., 2016). Within the first years of life, left ventricular dilation regresses and systolic function recovers in a substantial number of patients (Rigaud et al., 2013; Kang et al., 2016). In these patients LVEF remains largely stable (at ∼50%) (Spencer et al., 2006; Roberts et al., 2012). However, patients show a deficiency in increasing LVEF during exercise (Spencer et al., 2011). This is rather a clinical phenotype of heart failure with preserved ejection fraction (HFpEF) (Borlaug et al., 2006; Abudiab et al., 2013; Kraigher-Krainer et al., 2014) and contributes to the exercise intolerance of BTHS patients (Spencer et al., 2011).

Using the TAZ-KD mouse model, we recently discovered that CL deficiency also affected the integrity of the mitochondrial Ca^2+^ uniporter with implications on energy metabolism and redox homeostasis. Alterations in energetic demand due to increases in exercise need to be tightly matched by mitochondrial oxidative phosphorylation (Bertero and Maack, 2018). This is mediated by ADP-induced stimulation of respiration which results in accelerated oxidation of Krebs cycle-derived NADH and FADH2. Ca^2+^ transmission into mitochondria via the Ca^2+^ uniporter (MCU) stimulates Krebs cycle dehydrogenases to regenerate reducing equivalents (Cortassa et al., 2003). NADH levels are coupled to NADPH via the nicotinamide nucleotide transhydrogenase (NNT) reaction (Kembro et al., 2013; Nickel et al., 2014; Nickel et al., 2015). Via the glutathione reductase (GR), NADPH fuels the regeneration of glutathione (GSH), which is an important substrate for reactive oxygen species (ROS) defense mechanisms in the heart. Therefore, Ca^2+^-driven metabolic adaptation not only sustains both ATP production and NADH levels but also has a direct effect on ROS elimination under conditions of increased workload (Kohlhaas and Maack, 2010; Bertero and Maack, 2018).

Mitochondrial dysfunction in BTHS causes a substantial remodeling of cardiac metabolism including a reduction of fatty acid oxidation, which is the primary energy source to meet the cardiac energy demand (Kiebish et al., 2013; Chatfield et al., 2022; Chowdhury et al., 2023; Kutschka et al., 2023). Reduced fatty acid oxidation is compensated by an increase in glucose uptake. Activation of the integrated stress response signaling pathway directs glucose into the serine biosynthetic pathway and 1C metabolism and furthermore, activates the glutamate/cystine antiporter exchange (xCT) system, synergizing to generate glutathione for ROS defense (Kutschka et al., 2023). How this complex remodeling of metabolism itself affects lipid biogenesis is currently unknown.

A role in maintaining mitochondrial function has been ascribed also to other mitochondrial lipids, including phosphatidic acid (PA) and phosphatidylethanolamine (PE) (Chan and McQuibban, 2012; Joshi et al., 2012). Heart specific functions of the entire lipidome is reflected by a tissue-specific lipid composition including phosphatidylcholine (PC), PE, phosphatidylserine (PS), phosphatidylinositol (PI), phosphatidylglycerol (PG) and CL species in the heart (Pradas et al., 2018). Lipids also act as precursors for mediator molecules and obtain signaling functions (Tomczyk and Dolinsky, 2020). However, information about lipid profiles in cardiac tissue is limited in BTHS patients (Schlame et al., 2003). Although CL has been extensively studied in BTHS animal models, other lipid species and their composition have not been studied adequately (Byeon et al., 2021). A mouse model of tafazzin deficiency, which effectively mimics the major molecular and physiological features observed in BTHS patients, may provide a better understanding of how tafazzin deficiency affects cardiac lipid compositions and pathogenesis of BTHS. Therefore, we used the TAFAZZIN-knockdown mouse model to gain insights into lipid profiles and their compositions in cardiac tissue in BTHS.

In this study, we used ESI-MS/MS to measure a total of 193 individual lipid species in both 10-week-old and 50-week-old wild type (WT) and TAFAZZIN-knockdown mice (TAZ-KD). Considering that the genetic background of the mice significantly influences the observed phenotype (Wang et al., 2023), we carefully selected a mouse model that closely resembled the patient phenotype with diastolic dysfunction with preserved ejection fraction, and arrhythmic vulnerability (Bertero et al., 2021), or dilated cardiomyopathy that can lead to heart failure with reduced ejection fraction (Acehan et al., 2011). Interestingly, no ultrastructural alterations were reported in young mice, but functional impairments like slight diastolic dysfunction, whereas left ventricular ejection fraction and cardiac output were unchanged (Bertero et al., 2021). Ultrastructural mitochondrial and sarcomeric abnormalities and functional impairments with pronounced diastolic dysfunction and increased atrial natriuretic peptide as signs of heart failure were reported in older mice (Acehan et al., 2011; Bertero et al., 2021). The aim of the current study was to evaluate the lipid alterations and lipid composition in BTHS to gain a deeper understanding of its pathogenesis. By conducting a quantitative lipidome profiling analysis, we aimed to uncover crucial insights into the biological processes and cellular mechanisms in BTHS pathogenesis and ultimately improve our knowledge of disease development, progression, and treatment. Ultimately, this knowledge may contribute to the identification of novel therapeutic approaches based on our understanding of lipid profile changes in BTHS.

## 2 Materials and methods

### 2.1 Mice

For this study, we used a transgenic TAFAZZIN-knockdown (TAZ-KD) mouse model. Mice were obtained from Jackson Laboratories: (B6.Cg-Gt (ROSA) 26Sortm37 (H1/tet0-RNAi:Taz) Arte/ZkhuJ (stock No. 014648). The genetic background of these mice is C57BL/129S6 and contains a functional NNT. The knockdown is mediated by a TAFAZZIN short-hairpin-RNA (shRNA) that facilitates degradation of TAFAZZIN mRNA. Generation of the TAZ-KD mouse model has been described elsewhere (Acehan et al., 2011). Briefly, a TAFAZZIN shRNA expression cassette encoding for a shRNA against TAFAZZIN mRNA was inserted in the *ROSA26* acceptor locus on chromosome 6. The expression cassette contains a tetracycline-responsive element (Tet-On) in the promotor region. The Tet-On element allows expression of TAFAZZIN shRNA and depletion of TAFAZZIN mRNA upon feeding with doxycycline. Both WT and knockdown mice were fed with 625 mg/kg doxycycline containing chow beginning after fertilization. Before mating, doxycycline was withdrawn from female mice for 1 week and during mating to avoid male infertility. Doxycycline treatment was resumed upon successful mating (presence of copulatory plugs) and continued until the end of the experiment. The mice were kept in pathogen-free stables with 12 h light/darkness cycles and controlled temperature and humidity. The genotype was verified with PCR with tissue from the tail. For our experiments, the hearts of 10-week-old and 50-week-old mice were obtained. Since no gender-specific differences in cardiac function have been found *in vivo* (Bertero et al., 2021), both male and female animals were used for analysis. The study was approved and conducted under the regulations of the local animal ethics committees (AZ: 55.2.2-2535-804) and institutional guidelines.

### 2.2 Lipid extraction and sample preparation

The mice were sacrificed by cervical translocation, the abdomen and thorax were opened, and the cardiovascular system was flushed with phosphate buffered saline to remove the blood. The hearts were snap-frozen immediately in liquid nitrogen and stored at −80°C. For lipid analysis, 15 mg of each frozen left ventricle muscle sample were cut with a scalpel and placed in a sample vial. It was transported to the university hospital of Regensburg in dry ice for mass spectrometry analysis.

The following lipid species were added as internal standards: PC 14:0/14:0, PC 22:0/22:0, PE 14:0/14:0, PE 20:0/20:0 (di-phytanoyl), PS 14:0/14:0, PS 20:0/20:0 (di-phytanoyl), PI 17:0/17:0, lysophosphatidylcholine (LPC) 13:0, LPC 19:0, lysophosphatidylethanolamine (LPE) 13:0, ceramide (Cer) d18:1/14:0, Cer d18:1/17:0, D7 free cholesterol (FC), cholesteryl esters (CE) 17:0, CE 22:0, triacylglycerides (TG) 51:0, TG 57:0, diacylglycerides (DG) 28:0 and DG 40:0. The lipid extraction procedure was performed according to the method described by Bligh and Dyer (Bligh and Dyer, 1959). The tissue was homogenized (in a precellys bead-based homogenizer) in methanol/water (1:1) with 1% sodium dodecyl sulfate. The homogenate (a volume corresponding to 2 mg wet weight) was added to the extraction. For low mass resolution tandem mass spectrometry, it was dissolved in 7.5 mM ammonium acetate in methanol/chloroform (3:1, v/v). For high resolution mass spectrometry, it was dissolved in chloroform/methanol/2-propanol (1:2:4 v/v/v) with 7.5 mM ammonium formate.

**FIGURE 1:**
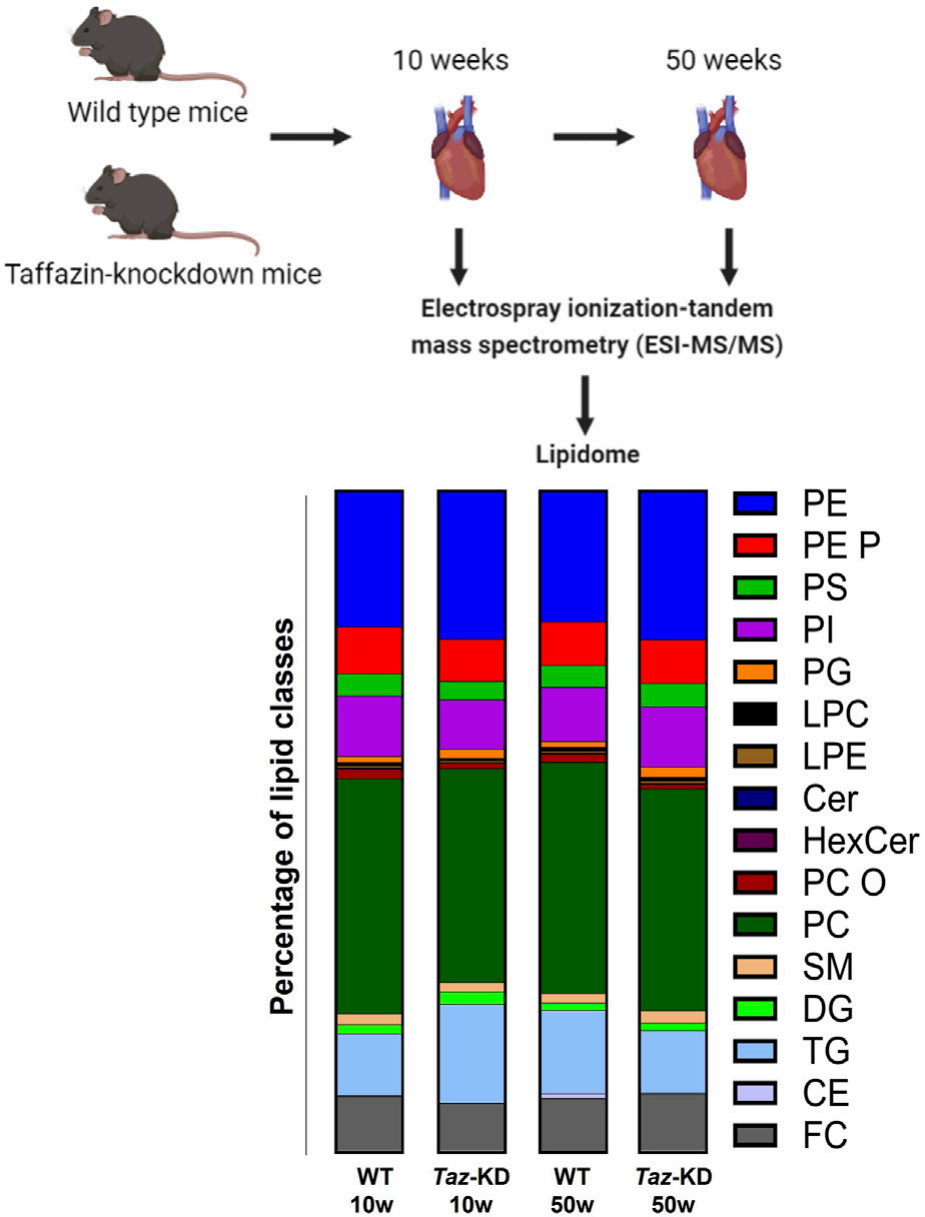
Experimental overview of the quantitative lipidomic analyses by mass spectrometry. The bar graph represents the percentage of lipid classes analyzed in this project.

In this study, the following lipid groups were analyzed: PC, ether-phosphatidylcholine (PC O-), LPC, PE, PE-based plasmalogens (PE P-), PI, sphingomyelins (SM), Cer, hexosylceramides (HexCer), CE, DG, and TG. An overview of the total lipid analysis of the study groups is shown in Figure 1. Lipid concentration is displayed on the species level or the molecular species level according to the updated LIPID MAPS classification system (Liebisch et al., 2020). For direct flow injection analysis (FIA), we used a triple quadrupole mass spectrometer (FIA-MS/MS; QQQ triple quadrupole) and a hybrid quadrupole-Orbitrap mass spectrometer for Fourier Transform Mass Spectrometry (FIA-FTMS; high mass resolution).

For FIA-MS/MS (QQQ), we used the positive ion mode, the setup and process are described elsewhere in detail (Liebisch et al., 2004). The following neutral losses were applied: PE 141, PS 185, PG 189, and PI 277 (Matyash et al., 2008). Analysis of PE P- was performed following to the principles described by Zemski-Berry previously (Zemski Berry and Murphy, 2004). For LPC, a fragment ion of m/z 184 was used (Liebisch et al., 2002) and for sphingosine based Cer and HexCer, a fragment ion of m/z 264 was used (Liebisch et al., 1999). Quantification was achieved by calibration lines generated by addition of naturally occurring lipid species to the respective sample matrix.

For measurement of TG, DG and CE with FTMS, the following conditions apply: FTMS in positive ion mode in range m/z 500–1,000 for 1 min, maximum injection time (IT) of 200 ms, an automated gain control (AGC) of 1 × 10^6^ *,* 3 microscans and a target resolution of 140,000 (at m/z 200). Recording of PC and SM was performed in range m/z 520–960. For measurement of [M + NH4]+ ions of free cholesterol (FC) (m/z 404.39) and D7-cholesterol (m/z 411.43), multiplexed acquisition (MSX) was used with the following settings: 0.5 min acquisition time, normalized collision energy of 10%, an IT of 100 ms, AGC of 1 × 10^5^, isolation window of 1 Da, and a target resolution of 140,000 (Horing et al., 2019). FIA-FTMS was quantified by multiplication of the spiked internal standard amount with analyte-to-internal standard ratio.

All lipid species annotations follow the latest proposal for shorthand notation of lipid structures derived from mass spectrometry (Liebisch et al., 2020). The annotation for QQQ glycerophospholipid species assumed the presence of only even numbered carbon chains. Final quantities of lipid species and total lipid were calculated and expressed in nanomoles per milligram of sample wet weight.

### 2.3 RT-qPCR

#### 2.3.1 RNA-isolation

For RNA isolation, the fresh murine tissue was snap-frozen in liquid nitrogen and were stored at −80°C before further use. The tissue was homogenized with 0.5 mL TriZol (Life technologies, Carlsbad, CA, United States) on ice. The lysate was transferred into a 1.5 mL reaction tube (Saerstedt, Nümbrecht, Germany) an incubated at room-temperature for 5 min, after that, 100 µL chloroform were added and incubated at RT for 3 min. Subsequently, the lysate was centrifuged 13.000 × g at 4°C for 15 min. The upper aqueous phase was transferred to a new tube and 250 μL Isopropanol were added. The lysate was incubated at room-temperature for 10 min. Subsequently, the lysate was centrifuged at 13.000 × g at 4°C for 10 min. After that, the supernatant was discarded, and the pellet was resolved in 0.5 mL ethanol 70% and centrifuged at 13.000 × g at 4°C for 5 min. After that, the supernatant was discarded, and the pellet was air-dried for 10 min. The pellet was solved in 10 μL DEPC-H₂O at 60°C for 10 min and then frozen at −20°C. RNA concentration was measured with a NANO-DROP™ 2000 (Thermo Scientific, Waltham, MA, United States). The RNA-samples were diluted to 1 μg/μL for DNAse-reaction. The DNAse-reaction was carried out using DNAse I, RNase free (Thermo Scientific) following the manufacturer’s instructions.

#### 2.3.2 cDNA synthesis

Reverse transcriptase reaction (RT-reaction) was conducted using Reverse transcription core kit (Eurogentec, Seraing, Belgium) following the manufacturer’s instructions. After reverse transcription, cDNA concentration was determined with NANO-DROP. The cDNA was diluted to 1 μg/μL for qPCR reaction.

#### 2.3.3 Quantitative polymerase chain reaction

DNA polymerase reaction was performed using a Takyon kit (Takyon™ No ROX SYBR 2X MasterMix blue dTTP, Eurogentec, Seraing, Belgium) following the manufacturer’s instructions. The primers for TAFAZZIN and mS12 were ordered from Eurofins (Ebersberg, Germany). TAFAZZIN primers: forward GAA​GTT​GAT​GCG​TTG​GAC​CC, reverse ACC​ATC​TCC​TCG​ACA​CAC​AG. mS12 primers: forward GAA​GCT​GCC​AAG​GCC​TTA​GA, reverse AAC​TGC​AAC​CAA​CCA​CCT​TC. The PCR reaction was carried out in a StepOnePlus Real-Time PCR System (Life sciences, applied biosystems, Waltham, MA, United States). Program used: 45 cycles of denaturation at 95°C for 15 s, annealing at 60°C for 60 s, and extension at 7°C for 1 min. Expression of TAFAZZIN was normalized to the level of mS12 and the comparative ∆∆CT method was used to evaluate gene expression.

### 2.4 Statistics

Two-way analysis of variance (ANOVA) and statistical comparisons between the groups via Tukey´s multiple comparisons post-test was calculated with GraphPad Prism 10.0.3 (GraphPad Software, California, United States). The graphs were created using the same software. The data is presented as mean ± standard deviation (SD) from control (*n* = 5) and TAZ-KD (*n* = 5) groups. A *p*-value of 0.05 or lower was considered as significant. Significance is indicated as *p ≤ 0.05, **p ≤ 0.01, ***p ≤ 0.001, ****p ≤ 0.0001.

## 3 Results

To gain insight into global changes in the cardiac lipidome in a mouse model of BTHS, we performed electrospray ionization tandem mass spectrometry (ESI-MS/MS) on hearts of the TAZ-KD and WT mice (Figure 1). Since aging affects the cardiac lipidome and leads to accumulation of specific lipids, mainly TGs in the heart (Eum et al., 2020), we analyzed 10- and 50-week-old tafazzin-deficient or WT control mice. Efficient TAFAZZIN-knockdown as confirmed by qPCR in both age groups. Relative gene expression in 10-week-old TAZ-KD mice was reduced in TAZ-KD hearts to 15% of the WT value. In 50-week-old TAZ-KD mice, relative TAFAZZIN gene expression was reduced to 4% of the WT value (Supplementary Figure S1).

In total, 193 individual lipid species were determined with ESI-MS/MS. We performed quantitative analysis of 9 PS, 9 PG, 7 LPE, 22 PC, 6 PC O, 7 LPC, 15 PE, 19 PE P, 13 PI, 12 SM, 6 HexCer, 6 Cer, 5 CE, 7 DG, and 50 TG lipid species from mouse heart homogenates (Figure 1). Tafazzin-deficiency clearly affected particular lipid classes including PC and PE and their ether derivatives and the lysophospholipids LPC and LPE. High variability in individual species composition was particularly found in the TG fraction. All lipid species that show no significant alterations are summarized in Table 1.

**TABLE 1:** List of analyzed lipid classes that show no statistically significant alterations between the analyzed groups.

PE	PE P	PS	PI	PG	LPC	LPE	Cer	HexCer	PC O	PC	SM	DG	TG	CE	FC
PE 38:1	PE P-16:0/16:0	PS 36:1	PI 34:2	PG 34:2	LPC 16:0	LPE 16:0	Cer 18:1; O2/20:0	HexCer 18:1; O2/16:0	PC O-34:3	PC 32:0	SM 34:1; O2	DG 34:1	TG 46:0	CE 20:4	
PE 38:2	PE P-16:0/18:1	PS 38:4	PI 36:1		LPC 18:0	LPE 18:0	Cer 18:1; O2/22:0	HexCer 18:1; O2/20:0		PC 32:1	SM 36:1; O2	DG 34:2	TG 46:1		
PE 38:6	PE P-16:0/18:2	PS 38:6	PI 36:2		LPC 18:1	LPE 18:1	Cer 18:1; O2/24:0	HexCer 18:1; O2/22:0		PC 34:1	SM 36:2; O2	DG 36:2	TG 48:0		
PE 40:5	PE P-16:0/20:3	PS 40:5	PI 36:3		LPC 20:4	LPE 18:2		HexCer 18:1; O2/23:0		PC 34:3	SM 38:2; O2	DG 36:3	TG 48:1		
PE 40:6	PE P-16:0/22:4	PS 40:6	PI 38:3			LPE 22:5		HexCer 18:1; O2/24:0		PC 35:1	SM 39:1; O2	DG 36:4	TG 48:2		
PE 42:7	PE P-16:0/22:5	PS 40:7	PI 40:4			LPE 22:6		HexCer 18:1; O2/24:1		PC 35:2	SM 39:2; O2	DG 38:6	TG 48:3		
	PE P-16:0/22:6		PI 40:5							PC 36:3	SM 40:1; O2		TG 49:1		
	PE P-18:0/22:4									PC 36:4	SM 42:1; O2		TG 49:2		
	PE P-18:0/22:5									PC 37:2			TG 50:1		
	PE P-18:0/22:6									PC 37:4			TG 50:2		
	PE P-18:1/18:2									PC 38:2			TG 50:3		
	PE P-18:1/22:4									PC 39:4			TG 50:4		
	PE P-18:1/22:5									PC 39:6			TG 50:5		
	PE P-18:1/22:6									PC 40:4			TG 51:1		
													TG 51:2		
													TG 51:3		
													TG 51:4		
													TG 52:2		
													TG 52:3		
													TG 52:4		
													TG 52:5		
													TG 52:6		
													TG 53:2		
													TG 53:3		
													TG 53:4		
													TG 53:5		
													TG 54:2		
													TG 54:3		
													TG 54:4		
													TG 54:5		
													TG 54:6		
													TG 54:7		
													TG 55:3		
													TG 55:4		
													TG 55:5		
													TG 56:3		
													TG 56:4		
													TG 56:5		
													TG 56:6		
													TG 56:7		
													TG 56:8		
													TG 58:5		
													TG 58:6		
													TG 58:7		
													TG 58:8		
													TG 60:8		
													TG 60:9		

### 3.1 Phosphatidylcholine and lysophosphatidylcholine

**FIGURE 2:**
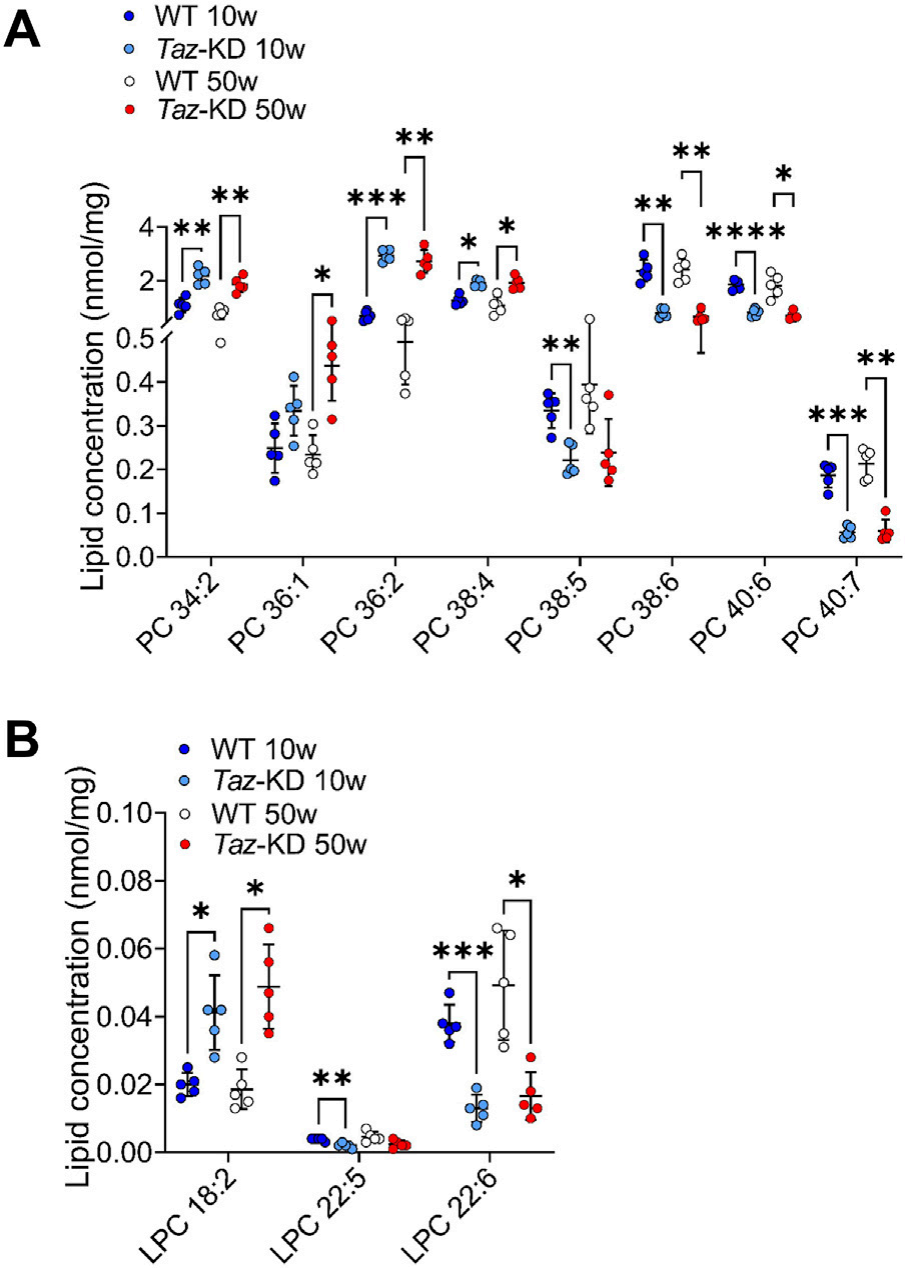
Significant changes of PC **(A)** and LPC **(B)** lipid species. Values are represented as nmol/mg of heart tissue. Values are presented as mean ± SD, *p*-value: *p < 0.05, **p < 0.01, ***p < 0.001, ****p < 0.0001. PC, Phosphatidylcholine; LPC, Lysophosphatidylcholine. WT, wild type; TAZ-KD, TAFAZZIN-knockdown.

PC species were previously analyzed in 4-month-old TAZ-KD mice, but a comprehensive analysis of the PC pool in aged animals has not been performed yet (Kiebish et al., 2013; Russo et al., 2022). 22 PC species were analyzed in the heart extracts. For better visualization, only significantly altered lipids are shown in Figure 2A. A genotype-specific distribution pattern was noted. The TAZ-KD group showed higher levels of short-chain PC species with 1–4 double bonds and reduced levels of long-chain PCs with 5–7 double bonds. Specifically, the levels of PC 34:2, PC 36:2 and PC 38:4 were found to be increased in the TAZ-KD groups at both 10 and 50 weeks of age, whereas the levels of polyunsaturated PC species with more than 5 double bonds (PC 38:6, PC 40:6, and PC 40:7) were reduced in the TAZ-KD groups. This pattern represents a shift in the TAZ-KD group with reduced long-chained poly-unsaturated PC species and increased levels of shorter-chained less-saturated PC species.

LPC are mainly produced by the enzyme phospholipase A2 but are also the product of the tafazzin mediated transacylation reaction of fatty acids from PC (Vreken et al., 2000; Xu et al., 2006). LPC are often referred to as an “eat me signal” when exposed on the cell surface during apoptosis to recruit phagocytic cells (Lauber et al., 2003). We analyzed 7 LPC lipid species and significant results are displayed in Figure 2B. We found genotype-specific alterations: Linoleic acid (LA, 18:2) containing LPC was increased in the TAZ-KD mice compared to the WT groups. Additionally, reflecting the decrease in long-chained polyunsaturated PC, docosapentaenoic acid (DPA) containing LPC and docosahexaenoic acid (DHA) containing LPC showed a significant approximately 2-fold reduction in the TAZ-KD groups when compared to the control groups (Figure 2B).

### 3.2 Phosphatidylethanolamine and lysophosphatidylethanolamine

**FIGURE 3:**
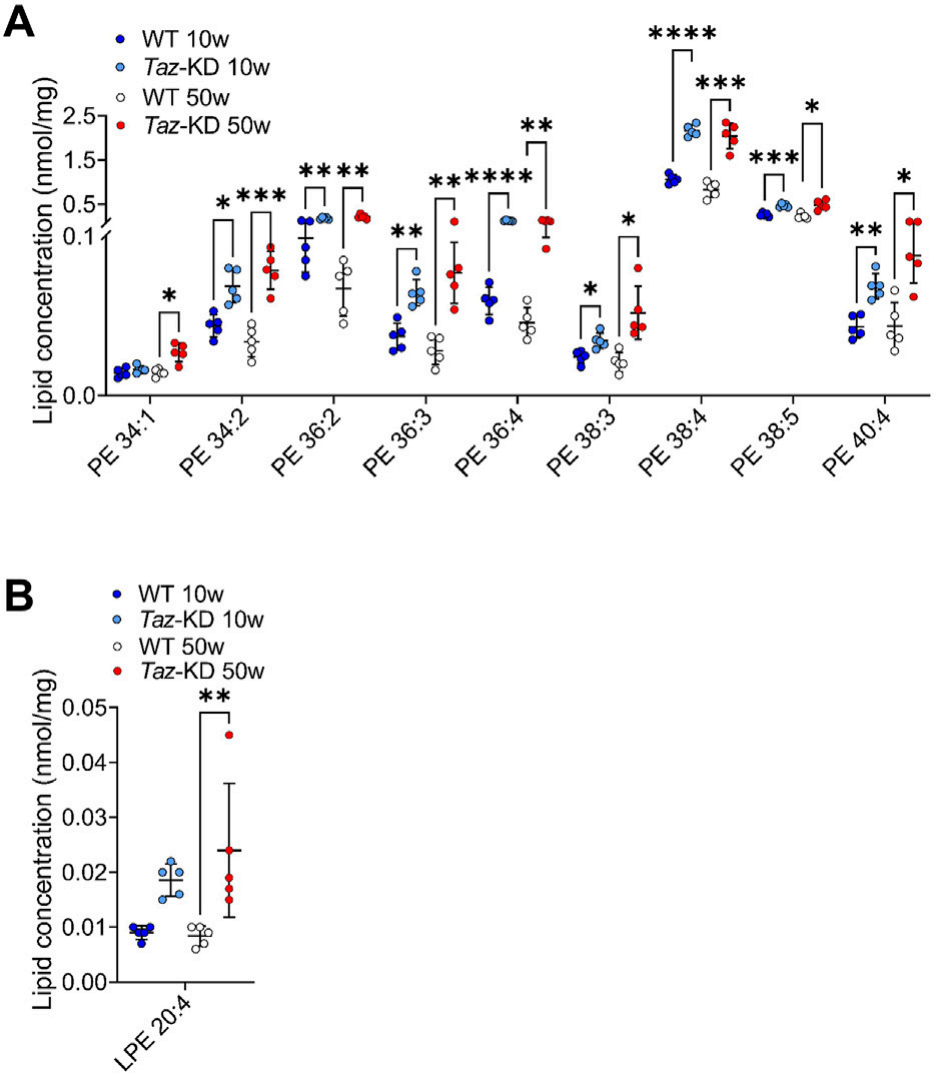
Significant changes of PE **(A)** and LPE **(B)** lipid species. Values are represented as nmol/mg of heart tissue. Values are presented as mean ± SD, *p*-value: *p < 0.05, **p < 0.01, ***p < 0.001, ****p < 0.0001. PE, Phosphatidylethanolamine; LPE, Lysophosphatidylethanolamine; WT, wild type; TAZ-KD, TAFAZZIN-knockdown.

Kiebish and others have reported a significant change in PE species, specifically an increase in lipid species containing arachidonic acid (AA) in 2-month-old TAZ-KD mice (Kiebish et al., 2013). In this study, we analyzed if these changes persist also in aged TAZ-KD mice. In total, 15 individual PE lipid species were measured, and significant changes are depicted in Figure 3A. We found alterations in all PE species except one (38:1), exhibiting a genotype-specific distribution pattern. The predominant long-chained polyunsaturated PE species with 2-5 double bonds (PE 38:3, PE 38:4, PE 38:5, PE 40:4, PE 40:5) were significantly elevated in the TAZ-KD groups compared to the corresponding WT group. Polyunsaturated PE species with higher number of double bonds were not resolved in our analysis. Only the mono-unsaturated species 38:1 showed no alterations (Figure 3A). Importantly, our study demonstrates that these findings are applicable not only to younger 10-week-old mice but also to older 50-week-old TAZ-KD mice.

LPE is a minor constituent of cell membranes, and the physiological significance of LPE is currently unknown. In this study, we quantified a total of 7 individual LPE species. We found an approximately 2-fold increase of AA containing LPE in TAZ-KD hearts. However, the higher abundant DHA containing LPE 22:6 did not exhibit a significant alteration (Figure 3B).

### 3.3 Phosphatidylserine, phosphatidylinositol and phosphatidylglycerol

**FIGURE 4:**
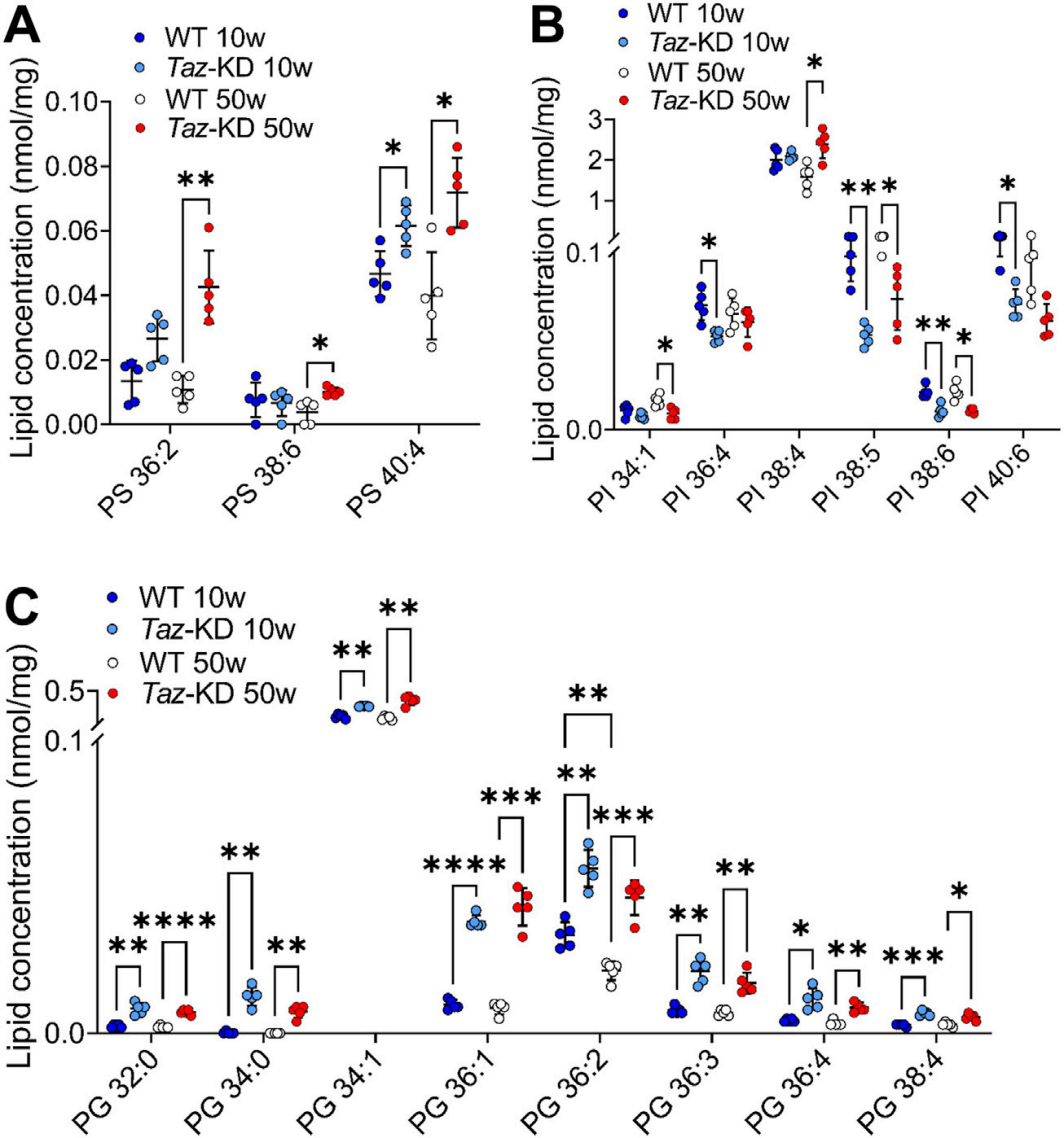
Significant changes of PS **(A)**, PI **(B)** and PG **(C)** lipid species. Values are represented as nmol/mg of heart tissue. Values are mean ± SD, *p*-value: *p < 0.05, **p < 0.01, ***p < 0.001, ****p < 0.0001. PS, Phosphatidylserine; PI, Phosphatidylinositol; PG, Phosphoglycerol; WT, wild type; TAZ-KD, TAFAZZIN-knockdown.

In CL deficient cells, the reduced conversion of PS by the CL-dependent PS decarboxylase (PISD) contributes to increased levels of PS (Seneviratne et al., 2019). We analyzed the individual composition of 7 PS. Among them, 3 PS species showed slight genotype-dependent alterations: The PS 40:4 displayed a slight but significant increase in the TAZ-KD hearts in comparison to WT mice groups, whereas PS 36:2 and PS 38:6 were only increased in older mice (Figure 4A). No statistical difference was observed in other PS species analyzed, particularly the highly abundant PS 40:6 showed no alterations.

PI plays a prominent role in the signal transduction from cell surface receptors to the nucleus (Balla et al., 2009). We analyzed a total of 13 PI species and displayed 6 altered lipid species in Figure 4B. Interestingly, we found a pattern of reduced PI levels in the TAZ-KD mice compared to the WT groups: PI 36:4, PI 38:5, PI 38:6, and PI 40:6 were notably reduced in the 10-week-old TAZ-KD hearts. However, the predominant PI 38:4 is increased in aged TAZ-KD hearts (Figure 4B).

PG constitutes approximately 1%-2% of total phospholipids and serves as an important precursor for the CL biogenesis. We analyzed the individual composition of 9 PG lipid species and found significant alterations in 8 species (Figure 4C). The PG species distribution exhibited a significant genotype-dependent pattern: We found a robust increase in all PG species, except for PG 34:2 in the TAZ-KD groups. It is remarkable that the predominant PG 34:1, which is more than 50-fold more abundant than all other PG species, displayed only a small but significant increase in the TAZ-KD groups (Figure 4C).

### 3.4 Ether-phosphatidylcholine (plasmanylcholine) and phosphatidylethanolamine-based plasmalogens

**FIGURE 5:**
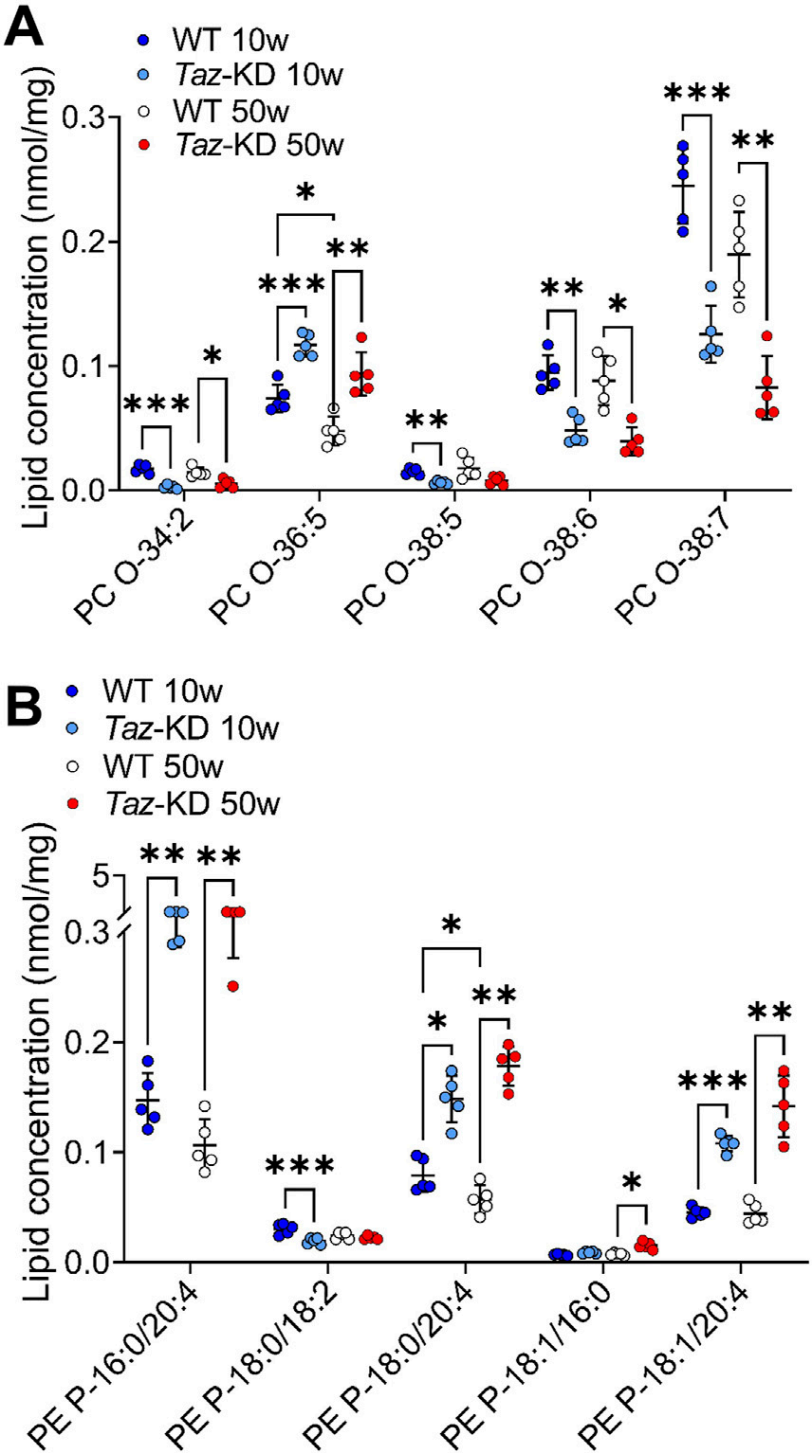
Significant changes of PC O- **(A)** and PE P- **(B)** lipid species. Values are represented as nmol/mg of heart tissue. Values are presented as mean ± SD, *p*-value: *p < 0.05, **p < 0.01, ***p < 0.001, ****p < 0.0001. PC O-, Ether-Phosphatidylcholine; PE P-, PE-based Plasmalogens; WT, wild type; TAZ-KD, TAFAZZIN-knockdown.

A significant decrease in the levels of PC O- species in the hearts of TAZ-KD C57BL/6N mice has been previously observed, using high resolution 31P nuclear magnetic resonance (NMR) (Kimura et al., 2018). However, a detailed analysis of PC O- species composition in young vs. old animals has not been performed. Therefore, we analyzed 6 PC O- species in both age groups and identified alterations in TAZ-KD hearts: The long-chain species PC O- 38:6 and PC O- 38:7 appeared reduced in the TAZ-KD groups to approximately half of the WT levels. Similarly, we found a decrease in PC O- 34-2 levels in the TAZ-KD groups. In contrast, a remarkable increase in PC O- 36:5 was present in the TAZ-KD groups, with levels approximately twice as high as the WT levels. Additionally, a significant age-dependent decrease of PC O- 36:5 was present in the WT group. (Figure 5A).

Given the substantial changes we observed in the group of PC O-, we sought to investigate whether PE P- species also underwent alterations. In total, we analyzed 19 PE P- species, of which 12 altered lipid species are depicted in Figure 5B. Our lipidomic analysis revealed significant alterations among the highly abundant PE P- species. Specifically, we found a consistent pattern of significantly increased levels of PE P- species containing AA in the TAZ-KD hearts: PE P- 16:0/20:4 was particularly increased, but also PE P-18:0/20:4, and PE P-18:1/20:4 species were increased compared to the control groups (Figure 5B). Moreover, PE P- levels are significantly reduced in 50 week old WT compared to 10 week old WT, this emphasizes the difference in the 50 week old group even more. Interestingly, DHA containing PE P- species appeared to be the predominant PE P- species in the mouse heart, but no significant alterations were detected among this specific PE P- species (Figure 5B).

### 3.5 Sphingomyelin, ceramide, and hexosylceramide species

**FIGURE 6:**
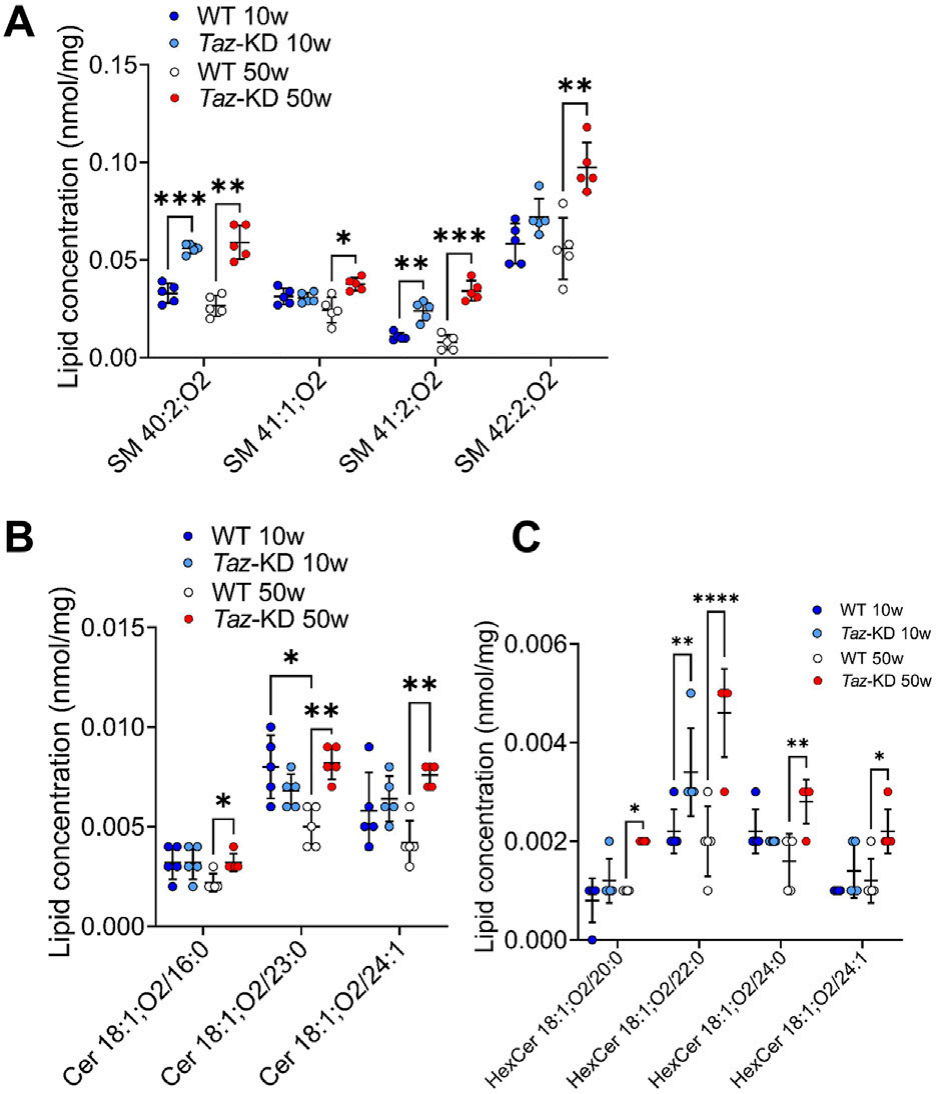
Significant changes of SM **(A)**, Cer **(B)** and HexCer **(C)** lipid species. Values are represented as nmol/mg of heart tissue. Values are presented as mean ± SD, *p*-value: *p < 0.05, **p < 0.01, ***p < 0.001, ****p < 0.0001. SM, Sphingomyelin; Cer, Ceramide; HexCer, Hexosylceramide; WT, wild type; TAZ-KD, TAFAZZIN-knockdown.

Sphingolipids play an important role in cell recognition and signal transduction. Interestingly, Cer have attracted great attention as a pathological increase of Cer levels is associated with heart failure (Zietzer et al., 2022). Within the sphingolipids, we analyzed the individual composition of 10 SM, 6 Cer and 6 HexCer species. Significant alterations are depicted in Figure 6. The very long-chained SM species 40:2; O2 and 41:2; O2 showed a notable significant increase in TAZ-KD hearts in both age groups. Furthermore, SM 41:1; O2 and SM 42:2; O2 were significantly increased only in the older mice hearts. Similarly, Cer species 18:1; O2/16:0, 18:1; O2/23:0, and Cer 18:1; O2/24:1 showed a significant increase only the in older TAZ-KD group. In part, this age dependent difference for Cer 18:1; O2/23:0 and Cer 18:1; O2/24:1 can be explained by a decrease in the old WT mice, the values for the TAZ-KD hearts and the young WT heart are similar. Among the analyzed 6 HexCer species, 4 species displayed significantly increased levels only in the 50-week-old TAZ-KD mice hearts (Figure 6C).

### 3.6 Free cholesterol, cholesteryl esters, triacylglyceride and diacylglyceride species

**FIGURE 7:**
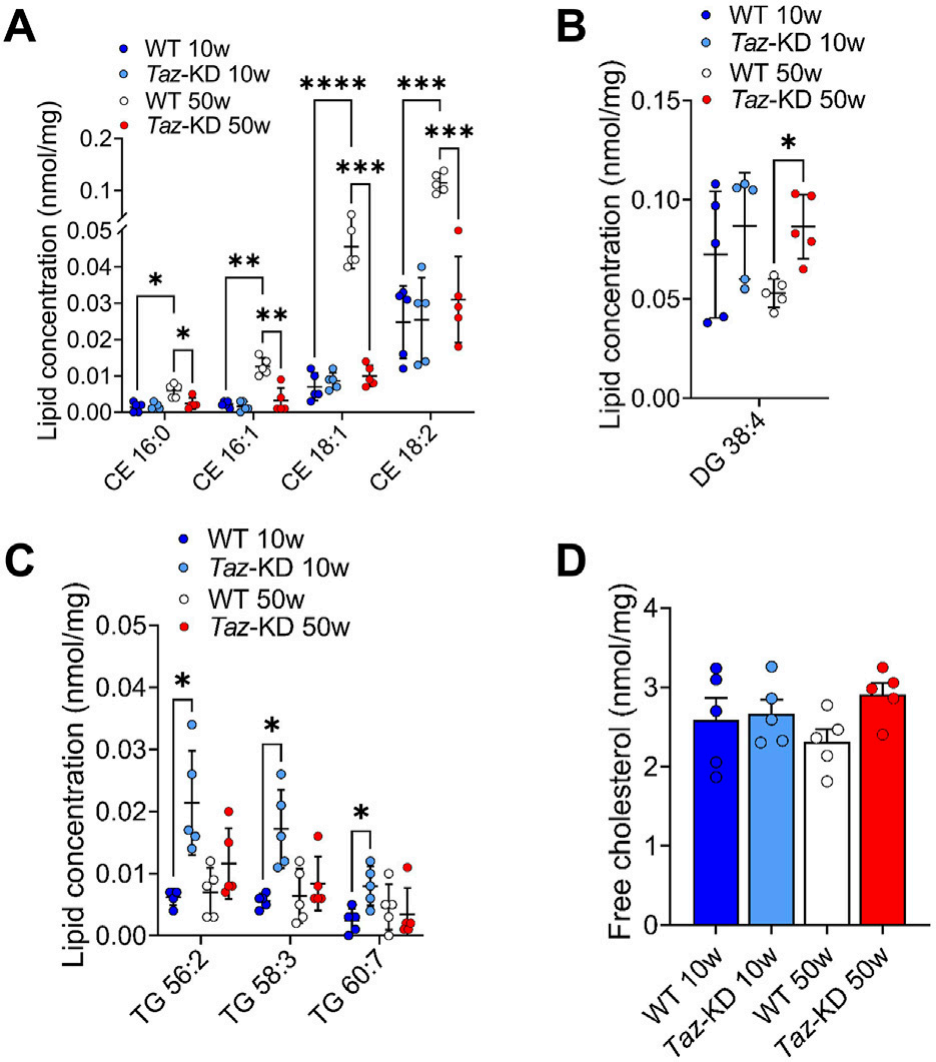
Significant changes of CE **(A)**, DG **(B)**, TG **(C)** and free cholesterol **(D)** lipid species. Values are represented as nmol/mg of heart tissue. Values are presented as mean ± SD, *p*-value: *p < 0.05, **p < 0.01, ***p < 0.001, ****p < 0.0001. CE, Cholesteryl ester; DG, Diacylglyceride; TG, Triacylglyceride; WT, wild type; TAZ-KD, TAFAZZIN-knockdown.

BTHS is associated with hypocholesterinaemia in patients (Barth et al., 1999), indicating a significant shift in cholesterol species composition due to tafazzin deficiency. Hauff and Hatch revealed that tafazzin-deficient lymphoblasts have reduced cholesterol synthesis capacities in response to increased demand, but basic cholesterol levels were found unaltered (Hauff and Hatch, 2010). Similarly, we observed free cholesterol at similar levels in all study groups (Figure 7D). In this study, we quantified 5 CE species. Significant changes were found in 4 species as shown in Figure 7A. There was a remarkable age-dependent increase in CE species in 50- compared to 10-weeks old WT. Interestingly, this elevation of CE species was not observed in the aging TAZ-KD mice (Figure 7A).

Lower myocardial FA extraction at rest and under exercise in BTHS patients, along with lower gene expression of fatty acid oxidation enzymes in TAZ-KD mice, might also affect fatty acid storage pools in the mouse heart (Cade et al., 2019; Cade et al., 2021; Chatfield et al., 2022). Therefore, we assessed the composition of 50 TG and 7 DG species. Among the TG species, TG 56:2, TG 58:3 and TG 60:7 displayed a slight increase in the 10-week-old TAZ-KD group compared to the WT groups (Figure 7C). No statistical differences were observed in the remaining TG species across all study groups. Regarding the DG species, all groups displayed a slight non-significant increase in the 10-week-old TAZ-KD hearts, whereas only DG 38:4 displayed a significant increase in aged TAZ-KD mice (Figure 7B). In conclusion, the observed increase in TG and DG content does not indicate a substantial increase in lipid storage in the heart due to the defect in mitochondrial lipid oxidation.

## 4 Discussion

BTHS is a rare and life-threatening metabolic disease known to cause mitochondrial dysfunction and cardiomyopathy due to altered CL metabolism (Han et al., 2005; Kiebish et al., 2013). Although CL loss and MLCL accumulation have been reported in several clinical and experimental studies (Schlame et al., 2003; Kiebish et al., 2013; Oemer et al., 2022), to date, there is very limited research on the involvement of other lipid metabolites in the pathology of BTHS. To our knowledge, this study is the first comprehensive quantitative lipidome analysis in heart tissue of the TAZ-KD BTHS mouse model using electrospray ionization-tandem mass spectrometry (ESI-MS/MS).

In this study, we evaluated how loss of tafazzin function affected the lipid composition in cardiac muscle. We analyzed the following lipid classes: PS, PG, LPE, PC, PC O, LPC, PE, PE P, PI, SM, HexCer, Cer, CE, DG, and TG from heart homogenates of WT and TAZ-KD mice. Our findings revealed distinct compositional alteration in major lipid species including PE, PC and PG in the TAZ-KD mice compared to the controls. The current study indicates only few changes in long chained and polyunsaturated PI and PS (Figure 4). 4 CE species (CE 16:0, 16:1, 18:1, and 18:2) showed a remarkable increase in the 50-week-old WT mice compared to 10-week-old WT groups (Figure 7A). This elevation of CE species was not observed in the aging TAZ-KD mice, which might be related to their leaner phenotype and smaller heart (Bertero et al., 2021).

Cardiac lipid profile is not only the result of cardiac lipid metabolism, but also influenced by other tissues and circulating lipoprotein composition. Lipoproteins are formed in the liver and transport nutritional lipids and lipids from hepatic synthesis to other tissues, e.g. Cer and sphingolipids (Iqbal et al., 2017). Thus, it strongly matters which organs are affected by tafazzin deficiency. The effect on the cardiac lipidome will probably vary between a cardiac-specific knockdown and a whole-body mutation like it is present in BTHS and in our TAZ-KD mouse model. Interestingly, Cole et al. could show in a 11-month-old TAZ-KD mouse model that tafazzin deficiency in the liver has totally different effects on the organ than cardiac tafazzin deficiency, probably because the CL species in liver are more diversified and the liver does not mainly depend on tetralinoleyl-CL. Liver functions of TAZ-KD mice are not impaired, the liver can even exert compensatory functions for the organism due to increased FAO. In contrast to the cardiac respiratory chain, liver respirasomes are normally organized in TAZ-KD liver leading to even reduced ROS-production (Cole et al., 2016).

Our analysis shows that tafazzin deficiency induces broad changes in diverse phospholipids. Besides CL, other phospholipids, such as PE are involved in mitochondrial integrity. Reducing mitochondrial PE levels in cell lines had dramatic consequences on mitochondrial morphology, assembly of the respiratory chain and ATP production and eliminating mitochondrial PE levels was embryonically lethal in mice (Steenbergen et al., 2006; Tasseva et al., 2013). A comprehensive understanding of lipid alterations is required to understand the molecular mechanism of disease pathology and to design strategies for therapy. Interestingly, mitochondrial phospholipids seem to be directly responding to nutritional lipid compositions, opening new avenues for therapeutic strategies. Experiments with TAZ deficient cell models, BTHS fibroblasts and induced pluripotent stem cells derived cardiomyocytes (iPSC-CM) revealed that the lipid composition of growth media strongly modulates CL composition (Valianpour et al., 2003; Wang et al., 2014; Oemer et al., 2021). Moreover, lipid supplementations not only affected CL, but also the levels of other phospholipids strongly dependent on the available lipid environment (Oemer et al., 2020). As the observed CL incorporation was independent on the presence of tafazzin, it was considered to be suitable for a therapeutic approach. Linoleic acid supplementation to a global and cardiac specific taffazin deficient mouse model revealed a delayed onset of cardiac phenotype, proving the concept of a therapeutical benefit of fatty acid supplementation (Zhu et al., 2024).

### 4.1 Phosphatidylethanolamine, phosphatidylcholine and phosphatidylglycerol

PE and CL are both essential for maintaining normal mitochondrial morphology and fusion, and it is suggested that PE may compensate for CL deficiency since studies in yeast have indicated that CL and PE may have overlapping functions in maintaining mitochondrial morphology and function (Joshi et al., 2012). Interestingly, PC species with 2–4 double bonds were significantly increased, while polyunsaturated PC species, containing more than 5 double bonds, showed a significant decrease in TAZ-KD compared to the WT mice (Figure 2A) in agreement with recent studies by other research groups (Kiebish et al., 2013; Zhu et al., 2021; Oemer et al., 2022; Russo et al., 2022). Consistent with our PC and PE results, Russo et al. showed that PC and PE species containing linoleic acid (LA, 18:2), such as PC 36:2 and PE 36:2, accumulated in 4-month-old TAZ-KD mice (Russo et al., 2022). Zhu et al. could show that this is also the case for TAFAZZIN-knockout mice (Zhu et al., 2021). Kiebish et al. also speculated that the increase in LA-containing lipids demonstrates the deficiency to incorporate LA in CL (Kiebish et al., 2013). Experiments with fatty acid supplementation to HEK cells revealed a specifically intensive exchange of externally added LA with CL species, indicating that LA is one of the most important fatty acids feeding into the CL pool (Oemer et al., 2022). However, it is important to note that Schlame et al. have reported different findings regarding PE and PC lipid species in the hearts of human patients. They observed a reduction in AA containing PE 18:0/20:4; while in our study, we found increased levels of AA containing PE 38:4 species in mouse hearts, similar to findings from 4-month-old TAZ-KD mice (Schlame et al., 2003; Russo et al., 2022). These discrepancies may be due to differences in lipid composition in human and mouse hearts or differences in the disease progression and severity between humans and mice. In our study, we found statistically significant alterations in almost all PG species except PG 34:2 in TAZ-KD hearts (Figure 4C). These data are in line with a study from Zhu et al. on a mouse model reporting on significantly increased levels of total PA and PG in cardiac tissue (Zhu et al., 2021).

### 4.2 Plasmalogens

Plasmalogens are members of a subclass of glycerophospholipids that have a vinyl ether linkage in sn-1 and an ester linkage in sn-2 position, playing crucial roles in neurochemical effects, cellular signaling, protection against ROS, and prevention of lipoprotein oxidation (Wallner and Schmitz, 2011; Kimura et al., 2019). In the context of BTHS, plasmalogens play a relevant role: The presence of a vinyl-ether bond makes plasmalogens highly susceptible to oxidation, for example via ROS. Therefore, plasmalogens may act as ROS scavengers, protecting other phospholipids, lipids and lipoprotein particles from oxidative damage, and play a critical role as endogenous antioxidants during states of increased oxidative burden (Brites et al., 2004). Mitochondria play a pivotal role in cellular redox metabolism, and structural remodeling of mitochondrial respiratory chain supercomplexes is thought to increase the propensity to form ROS (Maranzana et al., 2013; Bozelli et al., 2020).

Our study in TAZ-KD mouse hearts showed stable content of PE P- and even increased levels of AA containing PE P- species (PE P-16:0/20:4, PE P-18:0/20:4, and PE P-18:1/20:4) (Figure 5B). In contrast to our results, previous studies using high-resolution 31P-NMR demonstrated that in a cell and animal model of BTHS, plasmalogens level were significantly decreased (Kimura et al., 2018; Kimura et al., 2019). In this study, an increased plasmalogen turnover was assumed to be responsible for this effect: both plasmalogen synthesis and plasmalogen consumption processes are upregulated in TAZ-KD hearts. The plasmalogen synthesizing enzyme Far1 is strongly upregulated. At the same time, plasmalogen selective phospholipase iPLA_2_β is upregulated, releasing FAs at the sn-2 position and leading to reduced plasmenylcholine levels (Kimura et al., 2018). In the literature, increased ROS production has been reported in BTHS specimens (Chen et al., 2008; Bozelli et al., 2020). Plasmalogens have an antioxidant effect against a wide variety of reactive species. We speculate that the increased turnover of plasmalogens (Kimura et al., 2018) may be an endogenous antioxidant response against the increased oxidative load. Efforts have been made to promote plasmalogen biosynthesis to restore the low levels of PE P- and explore the effect of increased plasmalogen levels in BTHS cell models (Bozelli et al., 2020; Bozelli and Epand, 2022).

### 4.3 Arachidonic acid and docosahexaenoic acid containing lipids

AA (20:4) and DHA (22:6) are precursors for bioactive lipid mediators regulating inflammation, cell proliferation and cell differentiation. In our study, we found significantly higher levels of AA containing LPE in young TAZ-KD mice, while LPC 22:5, 22:6 species showed a significant reduction in TAZ-KD hearts (Figures 2B, 3B). Similarly, Russo et al. found decreased levels of LPC 22:6 in 4-month-old TAZ-KD mice (Russo et al., 2022). Increased levels of AA containing lipids were also found in the plasmalogen species PE P-16:0/20:4, PE P-18:0/20:4, and PE P-18:1/20:4 (Figure 5B). The altered CL pool can indirectly affect the metabolism of other lipid species, reflecting the complex interrelation of different lipid pools. It is known that CL activates the iPLA_2γ_-mediated hydrolysis of AA from PC (Liu et al., 2017). Interestingly, altered levels of DHA and AA in blood plasma and erythrocyte samples have been observed in a small cohort of BTHS patients (Barth et al., 2004). Deregulation of precursors of lipid mediators may link to alterations in the immune system in some BTHS patients. Overall, our results demonstrate significant impacts of tafazzin-deficiency on membrane biophysical properties, signaling, and overall lipid profile alterations, emphasizing the importance of disrupted lipid metabolism in triggering BTHS.

Increased levels of AA were particularly found in PE P- in TAZ-KD hearts. Similarly, Kiebish et al. reported increased levels of LA containing plasmalogen species (16:0/18:2, 18:0/18:2) in the TAZ-KD heart, whereas our study revealed unaltered levels at the age of 50 weeks, but even reduced levels of (18:0/18:2) in 10-week-old hearts (Figure 5B). Kiebish et al. reasoned that the increased levels of LA containing species culminate in increased AA species and their precursors (20:3 containing species). In synopsis, increased levels of AA containing plasmalogens could potentially also derive from 18:2 species (Kiebish et al., 2013).

### 4.4 Ceramides, sphingomyelins and hexosylceramides

Cer are essential precursors of various complex sphingolipids, including SM (Choi et al., 2021). Sphingolipids are localized in lipid bilayers and involved in numerous cardiac structural functions such as proliferation, differentiation, apoptosis, cell death, autophagy, and mitochondrial metabolism (Hannun and Obeid, 2008). Dysregulated sphingolipid metabolism has been linked to alterations in cardiomyocyte structure and function, with excessive accumulation of Cer and SM associated with dilated cardiomyopathy (DCM) (Guo et al., 2007; Sasset et al., 2016; Kovilakath and Cowart, 2020) and diabetic cardiomyopathy (Zlobine et al., 2016). HexCer accumulation is associated with ageing, and it also accumulates in human hearts after myocardial infarction (Trayssac et al., 2018; Samouillan et al., 2020). Conversely, inhibiting Cer biosynthesis in mice can improve characteristics of cardiometabolic disease (Cole et al., 2020; Wang et al., 2020; Choi et al., 2021). One mechanism connecting Cer to impaired cardiomyocyte function is their impact on mitochondria, where they disrupt energetics and induce apoptosis. Accumulated Cer in the inner mitochondrial membrane can impair respiratory capacity by interfering with electron transport chain activity (Park et al., 2008; Gaddam et al., 2011; Ji et al., 2017; Choi et al., 2021).

In our study, we detected a significant increase in very long-chained SM species in TAZ-KD mice and increased levels of long-chained Cer species in 50-week-old TAZ-KD mice (Figures 6A, B). Accumulation of several HexCer species in 50-week-old TAZ-KD hearts is consistent with previous findings from ischemic cardiomyopathy and the ageing heart, underlining the significance of HexCer accumulation in cardiomyopathy and ageing (Figure 6C) (Samouillan et al., 2020). This indicates genotype and age-dependent phenotype of HexCer, Cer and SM abundance in BTHS hearts and suggests their involvement in the development of BTHS cardiomyopathy.

## 5 Conclusion

Our current study provides a comprehensive and quantitative analysis of lipid classes and individual lipid species in TAZ-KD BTHS model at 10 and 50 weeks of age, using high-throughput tandem mass spectrometry. Our results reveal significant alterations in the myocardial lipidome due to tafazzin deficiency, affecting both young animals without a cardiomyopathic phenotype and older animals with heart failure. We highlight the importance of increased plasmalogen levels and the presence of bioactive lipid mediators, as evidenced by elevated AA and DHA containing lipid species. We provide first evidence of altered levels of SM, Cer, and HexCer, which are known to play a role in cardiovascular disease. This suggests new approaches for the diagnosis and therapy of BTHS by presenting new possible therapy targets that can be addressed by lipid supplementation therapies or lipid metabolism inhibition like plasmalogen and Cer metabolism.

## Figures and Tables

**Table:** 

**BTHS**	Barth Syndrome
**CL**	Cardiolipin
**DCM**	dilated cardiomyopathy
**LVEF**	left ventricular ejection fraction
**HFpEF**	Heart failure with preserved ejection fraction
**ROS**	Reactive oxygen species
**WT**	Wild type
**shRNA**	Short-hairpin-RNA
**TAZ-KD**	TAFAZZIN-knockdown
**GSH**	Glutathione
**GR**	Glutathione reductase
**FAO**	fatty acid oxidation
**AA**	Arachidonic acid
**PC**	Phosphatidylcholines
**PE**	Phosphatidylethanolamines
**PG**	Phosphatidylglycerols
**LPE**	Lysophosphatidylethanolamines
**LPC**	Lysophosphatidylcholines
**LA**	Linoleic acid
**SM**	Sphingomyelins
**Cer**	Ceramides
**HexCer**	Hexosylceramides
**PA**	Phosphatidic acid
**PS**	Phosphatidylserines
**PI**	Phosphatidylinositols
**PE P-**	Phosphatidylethanolamine-based plasmalogens
**DHA**	Docosahexaenoic acid
**DPA**	Docosapentaenoic acid
**PC O-**	Ether-phosphatidylcholines
**CE**	Cholesteryl esters
**TG**	Triacylglycerides
**DG**	Diacylglycerides

## Data Availability

The original contributions presented in the study are publicly available. This data can be found here: https://doi.org/10.6084/m9.figshare.25635084.v1.
