# Supplementary material for: Tafazzin deficiency causes substantial remodeling in the lipidome of a mouse model of Barth Syndrome cardiomyopathy
Source: Front Mol Med. 2024 Apr 29;4:1389456. doi: 10.3389/fmmed.2024.1389456 (PMC11285559; doi:10.3389/fmmed.2024.1389456)
Supplement: Supplementary file 1 [file Image1.pdf]

# Supplementary Figure

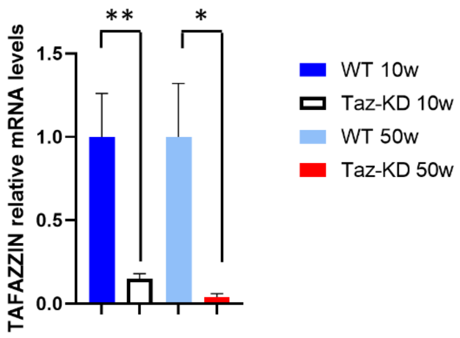


Supplementary Figure 1
RNA expression of TAFAZZIN expressed as a relative expression to the mitochondrial housekeeping gene mS12. WT 50w n=4, WT 10w n=5, TAZ-KD 10w n=6. Values are presented as mean ± SD, P-value: *P < 0.05, **P < 0.01.
